# A new cut-off value of FRAX tools as an osteoporosis screening tool for Thai geriatric population

**DOI:** 10.1038/s41598-025-90594-z

**Published:** 2025-02-22

**Authors:** Apichat Asavamongkolkul, Nath Adulkasem, Ekasame Vanitcharoenkul, Chandhanarat Chandhanayingyong, Panai Laohaprasitiporn, Krabkaew Soparat, Pojchong Chotiyarnwong, Aasis Unnanuntana

**Affiliations:** Department of Orthopaedic Surgery, Faculty of Medicine Siriraj Hospital, Mahidol University, 2 Prannok Road, Bangkoknoi, Bangkok, 10700 Thailand

**Keywords:** Osteoporosis, Screening, Fracture risk assessment tool, FRAX, Bone mineral density, Calcium and phosphate metabolic disorders, Health policy

## Abstract

Identifying osteoporosis in geriatric populations is essential for fragility fracture prevention. While dual-energy X-ray absorptiometry (DXA) remains the gold standard for diagnosing osteoporosis, its availability and cost for mass screening are limited. This study aims to determine an effective fracture risk assessment tool (FRAX) cut-off value for screening osteoporosis in the Thai geriatric population. The demographic data, FRAX hip fracture (HF), major osteoporotic fracture (MOF), and Bone mineral density (BMD) of community-dwelling Thai adults aged ≥ 60 years, conducted between March 2021 to August 2022 were analyzed. Osteoporosis is defined as a BMD T-score ≤ − 2.5. The accuracy of FRAX in identifying osteoporosis was assessed using the area under the receiver operating characteristic curve (AUC). Among 2991 participants (average age 69.2 ± 6.5 years), the discriminative ability was acceptable for both FRAX hip fracture (HF) (AUC = 0.75) and major osteoporotic fracture (MOF) (AUC = 0.72). A cut-off value of 1.5 for FRAX HF and 4.5 for FRAX MOF demonstrated excellent sensitivity (90.4%) and a high negative predictive value (89.7%) in osteoporosis detection. This study identifies FRAX cut-off values that can effectively screen for high-risk osteoporosis in the Thai geriatric population and suggests that FRAX could be a valuable tool for initial osteoporosis screening in Thai seniors.

## Introduction

Osteoporosis is a condition in which the structural properties of the human skeletal system, both micro-architectural and mineral density properties, are significantly compromised^1^. Currently, the gold standard for osteoporosis diagnosis remains the bone mineral density (BMD) measurement using a dual-energy X-ray absorptiometry (DXA) scan^2^. Although BMD evaluation is beneficial in the early detection of osteoporosis, the availability of DXA scans is limited in some areas^3^. Moreover, although the specificity is high, the sensitivity of a DXA scan in predicting fragility fractures is insufficient, making it unsuitable for population-based screening^4^. Therefore, most clinical practice guidelines advise DXA scans for BMD evaluation, mainly in high-risk populations. Nevertheless, the accuracy of these established criteria is not known. Accordingly, several evidence-based osteoporosis screening tools have been developed to enhance the accuracy of osteoporosis screening. Examples of these tools include the Simple Calculated Osteoporosis Risk Estimation (SCORE), the Osteoporosis Risk Assessment Instrument (ORAI), and the Osteoporosis Self-assessment Tool for Asians (OSTA)^5–7^. Unfortunately, these tools were not widely validated, which hindered the utilization and generalizability of these tools in clinical practice.

The Fracture Risk Assessment Tool (FRAX) is a well-known clinical prediction tool for fracture risk prediction^4,8^. While the FRAX tool has been used in Thailand since February 2010, it became more popular after 2016 since the national practice guidelines recommended using this tool^9^. FRAX utilized several clinical predictors, with or without BMD, to formulate the risk of fragility fracture, allowing clinicians to identify patients who are at risk of fragility fracture, both fragility hip fracture (HF) and major osteoporotic fracture (MOF)^8^. Several clinical practice guidelines recommend the use of FRAX results as an intervention threshold for osteoporosis treatment^10–14^. Nevertheless, some patients whose FRAX results didn’t exceed the intervention threshold demonstrated a degenerative change in skeletal structural properties^15–17^. Teeratakulpisarn et al. demonstrated that in some patients whose FRAX without BMD was low, the fracture risk became more significant after inputting the BMD value and might exceed an intervention threshold^17^. Accordingly, this group of patients could still benefit from further investigation and management, despite not having a substantial risk of fragility fracture evaluated solely from the clinical predictors^17^. Therefore, using FRAX to identify patients who might benefit from further BMD evaluation could assist clinicians in the early detection of osteoporosis since this tool has been widely validated and used across different populations^18–20^.

This study intended to establish an appropriate cut-off value of FRAX results to early detect high-risk patients with osteoporosis. The established definition can be used as an indication for further BMD evaluation in patients who have a high risk of osteoporosis.

## Results

A total of 2991 eligible participants, with an average age of 69.2 ± 6.5 years, were enrolled in the study. Among them, 889 (29.7%) received a diagnosis of osteoporosis, revealing not only advanced age and a higher proportion of females but also lower weight, shorter height, and significant history of fragility fractures. Interestingly, osteoporotic patients in this study revealed lower percentages of both current smoking and alcohol consumption (Table 1).

**Table 1:** Demographic data of osteoporotic and non-osteoporotic community-dwelling participants.

Demographic data	Total	Osteoporosis	Non osteoporosis	*p*-value
	*N* = 2991	*N* = 889 (29.7%)	*N* = 2102 (70.3%)	
FRAX item				
Age (years) (mean ± SD)	69.2 ± 6.5	71.8 ± 7.3	68.1 ± 5.8	< 0.001
Female (n, %)	1,888 (63.1)	710 (79.9)	1,178 (56.0)	< 0.001
Weight (kg) (mean ± SD)	58.3 ± 11.6	50.8 ± 9.9	61.5 ± 10.8	< 0.001
Height (cm) (mean ± SD)	155.9 ± 8.3	152.0 ± 7.5	157.5 ± 8.1	< 0.001
History of previous fragility fracture (n, (%))	367 (12.4)	129 (14.7)	238 (11.5)	0.017
Parent fractured hip (n, (%))	102 (3.5)	26 (3.0)	76 (3.7)	0.379
Current smoking (n, (%))	392 (13.3)	85 (9.7)	307 (14.8)	< 0.001
Glucocorticoids (n, (%))	82 (2.8)	23 (2.6)	59 (2.8)	0.807
Rheumatoid arthritis (n, (%))	23 (0.8)	8 (0.9)	15 (0.7)	0.647
Secondary osteoporosis (n, (%))	32 (1.1)	10 (1.1)	22 (1.1)	0.847
Consume alcohol ≥ 3 units per day (n, (%))	192 (6.5)	43 (4.9)	149 (7.2)	0.022
FRAX fragility hip fracture (mean ± SD)	2.50 ± 2.45	3.77 ± 2.91	1.96 ± 2.00	< 0.001
FRAX major osteoporotic fracture (mean ± SD)	6.40 ± 3.86	8.28 ± 4.23	5.61 ± 3.40	< 0.001
CCI (median (p25, p75))	3 (2, 4)	3 (2, 4)	3 (2, 3)	< 0.001
BMD (g/cm^2^) (mean ± SD)				
Lumbar spine	0.948 ± 0.195	0.751 ± 0.109	1.033 ± 0.160	< 0.001
Femoral neck	0.764 ± 0.139	0.633 ± 0.090	0.820 ± 0.116	< 0.001
Total proximal femur	0.849 ± 0.160	0.696 ± 0.109	0.914 ± 0.131	< 0.001

FRAX, Fracture risk assessment tool; CCI, Charlson Comorbidity Index; BMD, Bone Mineral Density.

**Fig. 1:**
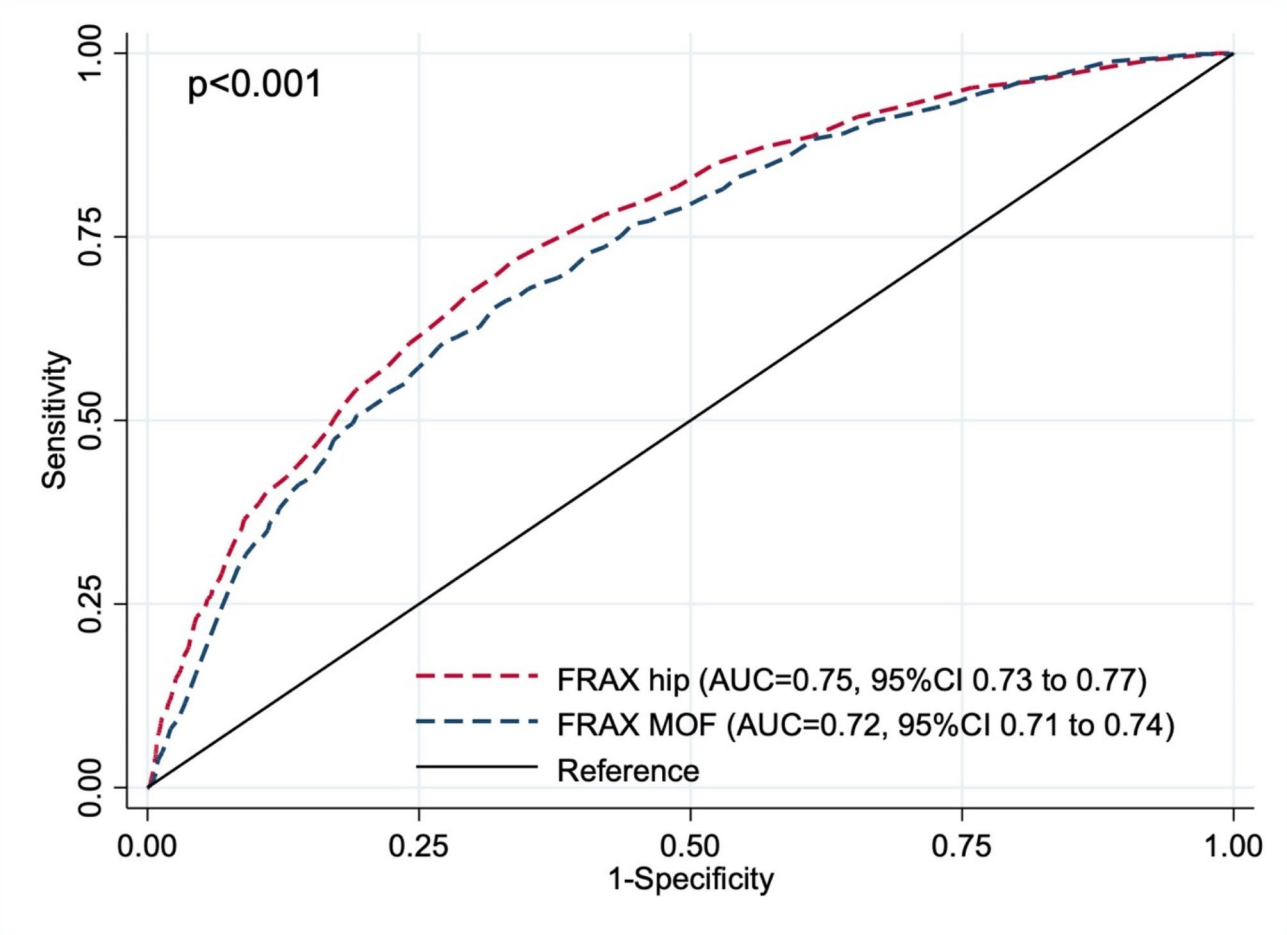
The area under the receiver operating characteristic curve (AUC) of osteoporosis screening accuracy between FRAX fragility hip fracture and FRAX major osteoporotic fracture.

As anticipated, osteoporotic patients presented elevated values for both FRAX HF and MOF. The overall discriminative ability for identifying osteoporosis was considered acceptable for both FRAX HF (AUC = 0.75, 95% CI 0.73 to 0.77) and FRAX MOF (AUC = 0.72, 95% CI 0.71 to 0.74) (Fig. 1). Detailed diagnostic indicators for osteoporosis diagnosis at various cut-off values are outlined in Table 2. The selection of the FRAX HF cut-off value of 1.5 and the FRAX MOF cut-off value of 4.5 was based on their sensitivity ≥ 80% while optimizing specificity. Consequently, participants with either FRAX HF ≥ 1.5 or MOF ≥ 4.5 were defined as high-risk patients in this study.

**Table 2:** Sensitivity, specificity, positive predictive value (PPV), and negative predictive value (NPV) of FRAX score for identifying osteoporosis.

FRAX value		Sensitivity (%)	95% CI	Specificity (%)	95% CI	PPV	95% CI	NPV (%)	95% CI
FRAX HF	1.0	93.2	(91.3–94.7%)	29.4	(27.5–31.4%)	35.8	(33.8–37.8%)	91.1	(88.6–93.1%)
	**1.5**	**81.8**	**(79.1–84.3%)**	**51.2**	**(49.0–53.4%)**	**41.4**	**(39.1–43.8%)**	**87.0**	**(85.0–88.8%)**
	2.0	71.7	(68.6–74.7%)	66.4	(64.3–68.4%)	47.4	(44.6–50.1%)	84.7	(82.9–86.5%)
	2.5	60.5	(57.2–63.8%)	75.9	(74.0–77.7%)	51.4	(48.3–54.5%)	82.0	(80.2–83.7%)
	3.0	50.2	(46.9–53.6%)	82.8	(81.1–84.4%)	55.2	(51.7–58.7%)	79.8	(78.0–81.4%)
	3.5	42.2	(38.9–45.6%)	87.2	(85.7–88.6%)	58.3	(54.3–62.1%)	78.2	(76.4–79.8%)
FRAX MOF	3.0	97.6	(96.4–98.5%)	14.8	(13.3–16.4%)	32.6	(30.8–34.4%)	93.6	(90.4–96.0%)
	3.5	93.4	(91.5–94.9%)	25.5	(23.6–27.4%)	34.6	(32.7–36.5%)	90.1	(87.4–92.4%)
	4.0	89.0	(86.8–91.0%)	36.0	(33.9–38.1%)	37.0	(34.9–39.1%)	88.6	(86.3–90.7%)
	**4.5**	**81.5**	**(78.8–84.0%)**	**47.0**	**(44.8–49.2%)**	**39.4**	**(37.1–41.7%)**	**85.8**	**(83.6–87.7%)**
	5.0	76.7	(73.8–79.5%)	55.3	(53.2–57.5%)	42.0	(39.6–44.5%)	84.9	(82.9–86.8%)
	5.5	70.2	(67.1–73.2%)	61.2	(59.1–63.3%)	43.3	(40.7–45.9%)	83.0	(81.0–84.8%)

95% CI, 95% Confidence interval.

Selected FRAX cut-off values for osteoporosis screening for Thais are in bold.

The proposed definition of high-risk patients underwent evaluation for diagnostic accuracy in osteoporosis screening. As a result, the FRAX-based definition demonstrated an excellent sensitivity of 90.4% (95% CI 88.3–92.3%) and an NPV of 89.7% (95% CI 87.4–91.7%) (Table 3).

**Table 3:** Diagnostic accuracy of the high-risk definition using the selected FRAX cut-off value.

High-risk definition (at least 1)	1. FRAX HF ≥ 1.5 2. FRAX MOF ≥ 4.5	
Diagnostic accuracy	Percentage	95% Confidence interval
Sensitivity	90.4	(88.3–92.3%)
Specificity	35.3	(33.2–37.4%
Positive predictive value	37.1	(35.0–39.2%)
Negative Predictive value	89.7	(87.4–91.7%)

Remarkably, when we calculated FRAX using both clinical factors and BMD results, we found that 18.4% of participants initially considered low-risk for fragility fractures actually had a risk exceeding the threshold for osteoporosis treatment.

## Discussion

This study identified appropriate cut-off values for the FRAX tool in osteoporosis screening. The findings showed that a FRAX HF value greater than 1.5 and a MOF value greater than 4.5 effectively identify patients at high risk for osteoporosis and could serve as screening criteria for BMD evaluation. Additionally, the proposed high-risk thresholds demonstrated a low false negative rate of approximately 10%, supporting their suitability for identifying patients who would benefit from further BMD assessment.

As expected, older age, female sex, less weight, shorter height, and history of previous fragility fractures were associated with osteoporosis^21^. These factors are well-known risk factors for osteoporosis and have been included in the FRAX tool^8^. While previous literature has shown that smoking and alcohol are also risk factors for osteoporosis, our study demonstrated an unexpected inverse association between these factors and osteoporosis^21^. A possible explanation is the potential presence of concurrent comorbidities among patients with smoking and alcohol consumption habits^22^. Consequently, this patient group may have been unintentionally omitted from our study due to poor health status, potentially impacting the association between these factors. Nevertheless, further investigations are required to elucidate the effect of smoking and alcohol consumption on osteoporosis in the Thai population.

The general indications for BMD evaluation were postmenopausal women aged ≥ 65 and those with secondary osteoporosis^2,23^. Additionally, populations with certain risk factors, including low body mass index (BMI), a family history of osteoporotic fracture, and systemic glucocorticoid usage, are also indicated for BMD assessment^2,23^. In conjunction with these previously recommended criteria, the findings from this study could enhance the effectiveness of osteoporotic screening. Our suggested FRAX-based screening criteria allow physicians to identify osteoporosis in its early stages, particularly among individuals who didn’t meet the previously established criteria. Based on findings from the ROSE study, applying the FRAX MOF threshold of 15% could help clinicians identify patients who should undergo BMD evaluation. The outcomes of this evaluation can assist clinicians in decision-making for osteoporosis treatment, resulting in an overall 5-year fracture risk reduction ranging from 0.5 to 2%^24^. Besides, it can identify patients whose FRAX results would exceed the intervention threshold after applying the BMD value, even if their risk appears low when relying solely on clinical predictors. Moreover, a previous study found that the combination of clinical risk factors criteria and DXA evaluation showed the smallest incremental cost-effectiveness ratio (ICER) compared to single screening methods^25^. Therefore, we are confident that our proposed FRAX-based screening criteria could enhance the cost-effectiveness of osteoporosis screening.

Since the FRAX tool was originally designed to predict fracture risk rather than diagnose based on BMD, its diagnostic accuracy might not be as high as other alternative tools (e.g., OSTA, ORAI, and SCORE)^18^. However, most of these tools were narrowly validated, which limited their generalizability. For instance, OSTA focused on the Asian population and has been validated in some Western countries, including the USA, Canada, Belgium, and the Netherlands^6,18^. Similarly, ORAI was developed using only the Canadian population and validated only in the USA, Belgium, the Netherlands, Japan, and Singapore^7,18^. Conversely, the FRAX tool had been widely validated using multiple cohorts with multi-nationalities, allowing broader use across different populations^18,20^. Furthermore, the FRAX calculator is user-friendly and easily accessible through web-based applications, allowing physicians to conduct osteoporosis screenings in clinical practice.

One significant concern of FRAX is its calibration to specific populations, which may not accurately represent fracture risks in other regions or ethnic groups. For instance, the U.S. FRAX calculator assigns lower fracture risks for Black, Asian, and Hispanic women compared to their White counterparts, potentially delaying necessary interventions in these groups^26^. The FRAX tool also excludes certain variables that are difficult for primary care practitioners to measure. These variables include physical activity, vitamin D deficiency, bone turnover markers, and loss of bone mass between sequential BMD measurements. Notably, falls are explicitly excluded despite their recognized role as a risk factor for fractures^27^.

The diagnostic accuracy of the FRAX tool in osteoporosis screening in this study is consistent with previous reports. Oka et al. found that a FRAX MOF of 7.2 had an AUC of 79% with a sensitivity of 82% and specificity of 63% among the Japanese population^28^. Similarly, Chandran et al. identified FRAX MOF of ≥ 3.7 (AUC = 77.8%) and HF of ≥ 0.5 (AUC = 79.6%) as appropriate thresholds for osteoporosis screening among Chinese, Malaysian, and Indian populations^29^. Conversely, the United States Preventive Services Task Force (USPSTF) strategy, which recommends BMD testing in postmenopausal women with a FRAX score of ≥ 9.3, demonstrated a lower performance of AUC at 56% with a sensitivity of 37.3% and specificity of 72.3% among Americans^30^. Although a higher FRAX score may provide better specificity for diagnosing osteoporosis, its lower sensitivity makes it less suitable as a screening criterion. Therefore, we advocate careful consideration of the appropriate cut-off FRAX value that maximizes the sensitivity while maintaining the specificity to minimize risks of osteoporosis under detection.

There are some limitations in this study. First, the findings of this study were derived from a cohort of healthy participants with independent ambulation. Consequently, the accuracy of our suggested screening criteria may not hold true for older adults with compromised health status. Second, the FRAX tool requires a relatively larger number of predictors, potentially limiting its practicality. Nonetheless, the FRAX tool is conveniently accessible through a web-based application using simple devices (e.g., personal computers and smartphones). A notable limitation is the absence of a longitudinal follow-up to ascertain whether the proposed cut-off points underestimate or overestimate fracture risk. This limitation arises from the cross-sectional design, which, while suitable for establishing baseline thresholds and associations, does not permit the direct observation of fracture outcomes over time. Future longitudinal studies are needed to validate these findings and assess their predictive accuracy. Finally, although the original FRAX tool has been widely validated, the diagnostic accuracy of our FRAX-based screening criteria demands external validity testing to ensure its generalizability regarding osteoporosis screening.

## Methods

This study performed a secondary analysis to evaluate the fracture risk of Thai community-dwelling older adults using a nationwide cross-sectional study conducted between March 2021 to August 2022. In summary, the original study focused on healthy Thai adults aged 60 years or older who were recruited from six distinct geographical regions of Thailand. A stratified multistage sampling technique was employed. Initially, the National Statistical Office divided the population into six strata based on Thailand’s regions (Northern, Northeastern, Central, Eastern, Western, and Southern). Each stratum comprised 500 participants. Subsequently, the National Statistical Office randomly selected two provinces from each region, followed by the random selection of ten enumeration districts in each selected province. Lastly, 25 eligible participants were randomly sampled from each enumeration district by village health volunteers.

The inclusion criteria required participants to be ambulatory and capable of undergoing central DXA for BMD assessment. The exclusion criteria included individuals who could not ambulate independently or who had conditions that contraindicated DXA scanning. During data collection, all participants were required to travel unassisted to the data collection point for a central DXA scan. Conditions that interfered with accurate DXA measurements included metallic implants in both the hip and lower spine regions or severe spinal deformities. The study protocol was approved by the Central Research Ethics Committee (CREC) of the National Research Council of Thailand (NRCT). The study has been performed in accordance with the Declaration of Helsinki.

### Data collection

Participants were appointed for data collection after written informed consent was obtained. Baseline characteristics, including age, gender, weight, height, and participants’ health status determined by the Charlson comorbidity index (CCI), were recorded. Variable required for FRAX evaluation, including a history of previous fragility fracture, parent fractured hip, smoking, glucocorticoids usage, rheumatoid arthritis, diagnosis of secondary osteoporosis, and alcohol assumption, were interviewed and recorded.

### Fracture risk evaluation

The fragility fracture risk was evaluated using the Fracture Risk Assessment Tool (FRAX) tool. In summary, the FRAX tool is a prediction tool that utilizes multiple clinical predictors, such as age, body mass index, and potential risk factors, to predict the 10-year probability of hip fracture (HF) and major osteoporotic fracture (MOF). We utilized the Thai-specific web-based FRAX calculator (accessible at https://frax.shef.ac.uk/FRAX/tool.aspx?country=57). This calculator was validated using data from multiple regions, including Khon Kaen (northeastern Thailand), Chiang Mai (northern Thailand), Songkhla (southern Thailand), and two university hospitals in Bangkok (central Thailand). A total of 1062 participants were involved in the validation, of whom 131 (12.3%) had a history of fractures (The data were obtained through personal communication with Professor Chatlert Pongchaiyakul, a key contributor to the development of the Thai dataset for the FRAX tool). In addition, the BMD value can be optionally applied to improve the accuracy of the prediction. The present study evaluates both FRAX HF and FRAX MOF without applying BMD value to determine the baseline fragility fracture risk of each participant. Subsequently, the FRAX prediction was re-calculated with an input of BMD value to determine the added prediction benefits of BMD in the FRAX tool. An intervention threshold was defined according to the American Association of Clinical Endocrinologists (AACE) guidelines, with 3% for HF or 20% for MOF^2^.

### Diagnosis of osteoporosis

The diagnosis of osteoporosis in the present study was defined according to the WHO recommendation based on BMD evaluation. All participants were evaluated for BMD at the lumbar spine (L_1_-L_4_), femoral neck, and total proximal femur using the mobile DXA scanner (Lunar Aria™; GE Healthcare, Chicago, IL, USA). The DXA scanner underwent regular quality assurance procedures following the International Society for Clinical Densitometry (ISCD) recommendation, demonstrating a coefficient of variation (CV) of 0.37%. The diagnosis of osteoporosis was made when a measured T-score was ≤ -2.5 SD from at least one site using an Asian BMD reference regarding the Thai Osteoporosis Foundation (TOPF) recommendation^23^.

### Statistical analysis

All statistical analyses were performed using STATA 18 (StataCorp LLC, College Station, TX, USA). Data normality was tested using the Shapiro-Wilk test. Means ± standard deviations (SD) are used to describe the continuous data with normal distribution. Conversely, continuous data with non-normal distribution are illustrated with medians and interquartile ranges. Categorical data are demonstrated using numbers and percentages. An independent *t*-test was used to determine the inferential statistic of normally distributed continuous data, while the Mann-Whitney U test was used for nonnormally distributed continuous data. The difference in categorical data was determined using Fisher’s exact test.

The overall discriminative ability of both FRAX HF and FRAX MOF in distinguishing osteoporosis was determined by the area under the receiver operating characteristic curve (AUC). Diagnostic accuracy indicators, including sensitivity, specificity, positive predictive value (PPV), negative predictive value (NPV), and their 95% confidence interval, were determined at each cut-off value. Subsequently, the FRAX value, both HF and MOF, that maximizes the sensitivity while preserving the specificity was selected as a screening cut-off value for osteoporosis. Patients whose FRAX HF or FRAX MOF exceeded the selected cut-off value were defined as high-risk patients in this study. Finally, the diagnostic accuracy of the proposed definition of high-risk patients was tested.

## Conclusions

This study demonstrated that the FRAX thresholds of 1.5 for HF and 4.5 for MOF could accurately identify osteoporosis among community-dwelling older adults. Our proposed FRAX-based osteoporosis screening criteria can serve as an additional indication for further BMD evaluation in patients with a high risk of osteoporosis. Moreover, the suggested screening criteria are simple and easy to use, making them a practical choice for cost-effective osteoporosis screening.

## Data Availability

The data that support the findings of this study are available from the corresponding author upon reasonable request. The data are not publicly available due to privacy or ethical restrictions.
